# Health Benefits of Nut Consumption in Middle-Aged and Elderly Population

**DOI:** 10.3390/antiox8080302

**Published:** 2019-08-12

**Authors:** Marius Emil Rusu, Andrei Mocan, Isabel C. F. R. Ferreira, Daniela-Saveta Popa

**Affiliations:** 1 Department of Pharmaceutical Technology and Biopharmaceutics, Faculty of Pharmacy, “Iuliu Hatieganu” University of Medicine and Pharmacy, 8 Victor Babes, 400012 Cluj-Napoca, Romania; 2 Department of Pharmaceutical Botany, Faculty of Pharmacy, “Iuliu Hatieganu” University of Medicine and Pharmacy, 8 Victor Babes, 400012 Cluj-Napoca, Romania; 3 Laboratory of Chromatography, ICHAT, University of Agricultural Sciences and Veterinary Medicine, 400372 Cluj-Napoca, Romania; 4 Centro de Investigação de Montanha (CIMO), Instituto Politécnico de Bragança (IPB), Campus de Santa Apolónia, 5300-253 Bragança, Portugal; 5 Department of Toxicology, Faculty of Pharmacy, “Iuliu Hatieganu” University of Medicine and Pharmacy, 8 Victor Babes, 400012 Cluj-Napoca, Romania

**Keywords:** tree nuts, peanuts, middle-aged population, antioxidants, age-related diseases, elderly, nut-enriched Mediterranean diet, inflammation, oxidative stress

## Abstract

Aging is considered the major risk factor for most chronic disorders. Oxidative stress and chronic inflammation are two major contributors for cellular senescence, downregulation of stress response pathways with a decrease of protective cellular activity and accumulation of cellular damage, leading in time to age-related diseases. This review investigated the most recent clinical trials and cohort studies published in the last ten years, which presented the influence of tree nut and peanut antioxidant diets in preventing or delaying age-related diseases in middle-aged and elderly subjects (≥55 years old). Tree nut and peanut ingestion has the possibility to influence blood lipid count, biochemical and anthropometric parameters, endothelial function and inflammatory biomarkers, thereby positively affecting cardiometabolic morbidity and mortality, cancers, and cognitive disorders, mainly through the nuts’ healthy lipid profile and antioxidant and anti-inflammatory mechanisms of actions. Clinical evidence and scientific findings demonstrate the importance of diets characterized by a high intake of nuts and emphasize their potential in preventing age-related diseases, validating the addition of tree nuts and peanuts in the diet of older adults. Therefore, increased consumption of bioactive antioxidant compounds from nuts clearly impacts many risk factors related to aging and can extend health span and lifespan.

## 1. Introduction

Healthy diet, along with physical and cognitive activity, is a modifiable lifestyle factor that has been associated with overall health in old age. Aging is a collective, multifactorial progression of physiological dysfunctions at cellular and tissue levels, as well as the deregulation of normal cell pathways and lost synchronicity among defense mechanisms in the body, leading to the development of age-related diseases [1].

A number of studies explored the phytochemical composition, bioactivity, and antioxidant potential of nuts [2,3]. Scientific evidence demonstrated the potential benefits of a higher intake of nuts or nut-enriched Mediterranean diets (MDs) against risk factors associated with pathological conditions linked with aging, such as cardiometabolic diseases, cancer, or cognitive disorders [4,5,6,7,8]. This review focused on middle-aged and older adults, a constantly growing group, with particular attention given to how these individuals can benefit from healthy dietary plans enhanced with tree nuts and peanuts, and how the extra bioactive antioxidant compounds may add quality to the increased life expectancy.

The aim of this study was to present up-to-date evidence confirming that nut intake and nut-enriched MDs [9] can be beneficial in delaying aging and can support modern medicine in treating age-related diseases. We investigated human studies that assessed the effects of nut consumption on the health of a middle-aged and elderly population 55 years of age and older. We searched PubMed/MEDLINE and EMBASE databases for peer-reviewed articles published in the decade from 2009 to June 2019. We also examined studies found in the references of meta-analysis that were in accordance with our inclusion criteria: subject age ≥55 years and nuts with antioxidant and anti-inflammatory activities added in the diet. In this review, the word “nuts” includes tree nuts, such as walnuts (*Juglans regia*), almonds (*Prunus dulcis*), hazelnuts (*Corylus avellana*), pistachios (*Pistacia vera*), pecans (*Carya illinoinensis*), cashews (*Anacardium occidentale*), pine nuts (*Pinus pinea*), Brazil nuts (*Bertholletia excelsa*), macadamias (*Macadamia integrifolia*), and also peanuts (*Arachis hypogaea*), botanically classified as legumes but with a nutrient profile comparable to tree nuts.

In addition, we summarized several nut phytochemicals with biological antioxidant activity, their possible mechanisms of actions, as well as the influence of nuts on gut microbiota (GM).

## 2. Association between Nut Consumption and Cardiometabolic Disorders

Globally, cardiometabolic diseases including type 2 diabetes mellitus (T2DM), coronary heart disease (CHD), coronary artery disease (CAD), and stroke are leading causes of morbidity and mortality [10]. Most of these diseases could be prevented by changing behavioral risk factors such as suboptimal diet [11].

Tree nuts and peanuts, because of their healthy antioxidant biochemical profile, can improve the lipid profile, increase insulin sensitivity and metabolism, and favorably influence other cardiometabolic risk factors [12].

### 2.1. Nut Consumption in Cardiometabolic Morbidity and Mortality

Clinical trials and lengthy prospective studies, focusing on men and women over the age of 55, showed that higher weekly nut intake can lower all causes and cause-specific morbidity and mortality [13,14,15,16,17,18,19,20,21] (Table 1).

Subjects at high cardiovascular (CV) risk, who supplemented their MD with 30 grams of tree nuts per day, at any point in time during the 4 years study period, had a 53% lower diabetes incidence compared to the control group [13]. Pan et al. [14] observed that women in the highest nut serving group were 26% less likely to develop diabetes than participants in the very low nut intake group, with 95% confidence that the true value is lying between 16%–35%. Similarly, when comparing high to very low nut intake, the prevalence of diabetes, obesity, and MS were 13%, 39%, and 26% less likely, respectively [15].

A single meta-analysis [22] confirmed that subjects in the highest total nut intake group had 32% less risk of dying from diabetes than those in the lowest group (relative risk, RR = 0.68, 95% confidence interval, CI: 0.52–0.90).

One prospective study showed a nonsignificant reduction in cardiovascular disease (CVD) mortality when comparing the highest with the lowest nut intake population groups [17]. However, two others concluded that participants who were fed nut-enhanced MDs had a significantly lower risk for CVD and CHD, as well as lower CVD, CHD, and all-cause mortality compared to the no-nut group [16,18]. Participants who consumed walnuts ≥1 serving per week had 19%, 21%, and 17% lower risk for CVD, CHD, and stroke, respectively, while those who consumed walnuts >3 servings per week had 47% lower risk of CV mortality compared with subjects who did not eat walnuts [18]. A very recent prospective study demonstrated lower CVD incidence (hazard ratio, HR = 0.83, 95% CI: 0.71–0.98) and CHD incidence (HR = 0.80, 95% CI: 0.67–0.96), 31% lower all-cause mortality and 34% lower CVD mortality for at least 5 servings of nuts per week compared to less than one per month [21]. In the same study, total nut intake was not significantly associated with stroke incidence. Moreover, a recent intervention study showed that individuals at high CV risk had a lower incidence of major CV events (myocardial infarction, stroke, death from CV causes) when nuts were added daily to MD compared with a low-fat, nut-free control diet [20]. Also, three or more servings of nuts per week, as compared to none, can even lower the risk of atrial fibrillation and heart failure [19].

Peripheral arterial disease (PAD), often caused by atherosclerosis, can lead to heart attack and stroke. A large cross-sectional study on mature adults indicated that daily nut consumption was associated with lower odds of PAD (OR = 0.79; 95% CI: 0.77–0.80, *p* < 0.001) compared to participants with the lowest intake of nuts [23].

A very recent meta-analysis of 11 observational studies confirmed that tree nut (but not peanut) consumption was negatively associated with metabolic syndrome (MS) (*p* = 0.04) [24]. Another meta-analysis showed a significantly reduced risk for CVD and CHD for higher nut consumption (including peanuts) [22]. Luu et al. [24] showed that in different ethnic groups, nut consumption was associated with decreased overall mortality and CVD mortality and that a higher versus lower quintile of nut intake had a statistically significant inverse association for ischemic heart disease (IHD). Mayhew et al. [25], after reviewing several large prospective cohort studies, concluded that nut consumption was inversely associated with total CVD, CVD mortality, total CHD, CHD mortality, and sudden cardiac death. While one meta-analysis established that daily nut intake can reduce the risk of stroke [26], another study did not find a significant association with stroke, but indicated an inverse association with IHD, overall CVD, and all-cause mortality for nut consumption [27]. A meta-analysis of randomized controlled trials (RCTs) and observational studies identified that nut feeding was inversely linked with nonfatal and fatal IHD and diabetes, but not stroke [28]. However, a meta-analysis of prospective cohorts to evaluate the relation between nuts and stroke risk and mortality, reported that nut consumption was inversely associated with stroke risk (RR 0.90, 95% CI: 0.83–0.98) and stroke mortality, when comparing the highest with the lowest nut intake [29].

High blood pressure (BP) is a major risk factor for CVD. The previous feeding studies showed that tree nuts or peanuts had no effect on BP. However, a very recent RCT on an elderly cohort (age, 69 years) proved that walnuts (42.5 grams per day) reduced systolic BP in the walnut group (−4.61 mm Hg, 95% CI: −7.43 to −1.79 mm Hg) compared to the no-walnut group (−0.59 mm Hg, 95% CI: −3.38 to 2.21 mm Hg)(*p* = 0.051) [30]. As no changes in diastolic BP were noticed, it is safe to say that walnut intake can reduce systolic BP in mature subjects, mainly in those with mild hypertension.

### 2.2. Nut Consumption and Blood Lipids

The effects of tree nut and peanut consumption on lipid profiles from intervention studies published in the last ten years [31,32,33,34,35,36,37,38,39,40,41,42,43] are summarized in Table 2. The results of these clinical trials in a middle-aged population indicate a causal association between higher nut intake and lower levels of total cholesterol (T-C), low density lipoprotein-cholesterol (LDL-C), non-high density lipoprotein-cholesterol (non-HDL-C), triglycerides (TG), and apolipoprotein B (apoB), all markers of CV morbidity and mortality.

After a 12 week trial and daily intake of 56 g almonds, compared with a no-nut control diet, plasma apoB, apoB/apoA-1 ratio, T-C, LDL-C, and LDL-C/HDL-C ratio decreased significantly by 15.6%, 17.4%, 6.0%, 11.6%, and 9.7%, respectively, in patients with T2DM [31]. Similarly, the inclusion of almonds in the diet of patients on chronic statin therapy revealed a 4.9% reduction in non–HDL-C compared with the no-nut group, and non-statistical significance decreases in LDL-C and TG [34]. Consumed before breakfast, 10 g almonds proved to increase serum HDL-C by 15% after 12 weeks in CAD patients with low initial HDL-C [35]. A new trial, comparing almond snacks with isocaloric carbohydrate snacks, demonstrated that almond snacks can improve the serum T-C/HDL-C ratio in women but not in men, with no change in body weight (BW) or inflammatory biomarkers in overweight and obese adults with high T2DM risk [43]. The short 8 weeks trial might be the cause for the differential gender results.

Our findings are aligned with data from a meta-analysis conducted by Musa-Veloso et al. [44] in which almond consumption was confirmed to significantly decrease T-C (*p* < 0.001), LDL-C (*p* = 0.001), and TG (*p* = 0.042), with no modification in HDL-C (*p* = 0.207). Also, Nishi et al. [45] showed that the daily consumption of almonds by middle-aged adults can improve the blood lipid profile, and a 3.5% decrease in the 10-year CHD risk was noticed for every 30 g increase in almond intake.

Consistent with the effects of other nuts, Brazil nuts and cashews can also improve lipid profiles. In a group of hypertensive and dyslipidemic subjects, the intake of partially defatted Brazil nuts significantly increased plasma selenium and the antioxidant activity of the glutathione peroxidase enzyme, and reduced oxidation in LDL-C compared to the baseline [37]. Mah et al. [39] demonstrated that adding cashews into the diet of a population with high LDL-C risk could lower the T-C, LDL-C, and LDL-C/HDL-C ratio. In agreement with these results, a very recent trial showed a significant decrease for the LDL-C/HDL-C ratio in a cashew diet group compared with a no-cashew control group [46].

Pistachio, a biochemically-rich tree nut, proved to have a lowering effect on CV risk factors. Daily pistachio intake significantly decreased the T-C and T-C/HDL-C ratio (*p* < 0.05), and TG levels (*p* = 0.003) compared to the control in T2DM adults [38]. Also, after 4 months of 57 g pistachio daily, small LDL particles and non-HDL particles significantly decreased compared to the nut-free diet [33]. This change of lipoprotein particle size may explain the decrease of CVD risk. Kay et al. [47] showed that the consumption of a pistachio-enriched diet, when compared to the control, significantly increased serum concentration of antioxidants, including beta-carotene, gamma-tocopherol, and lutein, and significantly decreased serum oxidized-LDL, an important factor in CVD.

Walnut, one of the most versatile tree nuts with its higher content of polyunsaturated fatty acids (PUFAs) including α-linolenic acid, and high antioxidant activity, may influence CVD risks via its lipid-lowering impact. Compared with a control diet without walnuts, a walnut-included diet for 6 months significantly decreased T-C and LDL-C and improved diet quality [36]. In a shorter cross-over trial, a walnut-enriched diet significantly reduced non-HDL-C (*p* = 0.025) and apoB (*p* = 0.009) compared with a control diet, while T-C displayed a tendency toward reduction (*p* = 0.073) [32]. Bamberger et al. [40] indicated that a walnut-enriched diet versus a control diet caused a significant decrease in fasting cholesterol (*p* = 0.002), LDL-C (*p* = 0.029), non-HDL-C (*p* ≤ 0.001), TG (*p* = 0.015), and apoB (*p* ≤ 0.001) in healthy mature adults. Also, 15 mL walnut oil daily (corresponding to ~28 g walnuts) added for 90 days to the diet of hyperlipidemic T2DM patients significantly decreased the T-C, LDL-C, T-C/HDL-C ratio (*p* < 0.001 for all), and TG level (*p* = 0.021), compared with the control group, while the HDL level showed an increased trend (*p* = 0.06) [48]. Similarly, Austel et al. [49] noticed beneficial changes in blood lipids after replacing saturated fats with walnut oil.

A meta-analysis of 26 trials confirmed that walnut-enriched diets compared with control groups significantly reduced T-C (*p* < 0.001), LDL-C (*p* < 0.001), TG (*p* = 0.03), and apoB (*p* = 0.008), with no significant modifications in BW or blood pressure [50].

Integrated into a typical Western diet, pecans, another tree nut with higher contents of PUFAs, showed a borderline significant lowering of T-C and LDL-C as compared to a nut-free control diet in overweight or obese adults with central adiposity [41].

A new trial confirmed that, compared to a carbohydrate control diet, adding 75 g per day of mixed nuts (tree nuts and peanuts) to healthy diets could significantly lower small LDL-C (*p* = 0.018), with a trend towards reduction for T-C (*p* = 0.066) and non-HDL-C (*p* = 0.067) in T2DM elderly patients [42].

These outcomes are validated by a meta-analysis of 61 trials which concluded that nut intake significantly lowered the levels of T-C, LDL-C, apoB, and TG, with the key factor of changing lipid profile appearing to be nut dose rather than nut type [51].

As many strategies for reducing T-C and LDL-C levels could lower HDL-C levels, all the dietary plans for lowering LDL-C levels should aim to maintain or even increase HDL-C. In their 2017 guidelines, the American Association of Clinical Endocrinologists and American College of Endocrinology recommend a minimum blood HDL-C level of 40 mg/dL in CVD risk individuals [52]. However, data [53] showed that small HDL particles present only a weak defense, the strong protection against CVD risks coming from large HDL units. Equally, small LDL particles, due to their proneness to oxidation compared with larger ones, are responsible for atherosclerosis progress and CVD, while large LDL components are only weakly linked with CVD [53]. Based on these many results, nut consumption can evidently aid in the management of a healthy lipid profile in mature adults.

### 2.3. Nut Consumption and Biochemical and Anthropometric Parameters

The outcome of tree nut and peanut consumption on biochemical and anthropometric parameters found in clinical trials published in the last ten years [31,41,42,54,55,56,57,58,59,60] are summarized in Table 3.

The trial of Li et al. [31] revealed that in Chinese T2DM patients, almond intake, compared with a control diet had a beneficial effect on glycemic control, lowering fasting insulin, fasting glucose, and Homeostatic Model Assessment—Insulin Resistance (HOMA-IR) by 4.1%, 0.8%, and 9.2%, respectively. The ingestion of a single serving (28 g) of almonds before a high-starch meal significantly reduced hemoglobin A1c (HbA1c) (*p* = 0.045) and postprandial glycemia (*p* = 0.043) in individuals with uncomplicated T2DM [54]. Another intervention study showed that almonds significantly decreased post-interventional fasting glucose by 5.9% (*p* = 0.01) and HbA1c by 3.0% (*p* = 0.04) as compared with that of control in T2DM subjects [59]. In the study of Hou et al. [60], the T2DM patients who replaced part of their starchy food with almonds or peanuts had decreased values for fasting blood glucose and 2-h postprandial blood glucose (*p* < 0.05) compared with the baseline. In addition, in the almond group a decrease in the HbA1c level from the baseline was found (*p* < 0.05) and none of the nut groups showed an increase of body mass index (BMI).

Several dietary interventions within the frame of the PREDIMED study tried to establish a relation between MDs supplemented with mixed nuts (walnuts, hazelnuts, and almonds) and biochemical or anthropometric parameters. In a clinical trial with high CV risk participants but no CVD at enrolment, data showed that MD supplemented with 30 g per day mixed nuts for 1 year significantly decreased waist circumference (Wc) compared to baseline, and also lowered LDL-C and shifted LDL size to a less atherogenic pattern [55]. Lasa et al. [56] indicated that a daily quantity of 30 g nuts (walnuts, hazelnuts, and almonds) added to MDs was associated with a significant reduction in BW (*p* = 0.021) compared with the control, with improved glucose metabolism in both the nut group and low-fat diet group. Rodríguez-Rejón et al. [58] concluded that the nut-enhanced MD lowered glycemic index and glycemic load, two indices that can be associated with T2DM and CHD.

An RCT conducted on patients with diabetes established that 75 g mixed nuts (tree nuts and peanuts) added to the diet for 3 months, besides improving the lipid profile, also significantly lowered HbA1c compared with a carbohydrate diet (*p* = 0.026) [42]. In another trial conducted on T2DM subjects, the addition of 15 mL walnut oil to the diet for three month significantly decreased HbA1c level by 7.86% (*p* = 0.005) and fasting blood glucose by 8.24% (*p* = 0.001) compared to control [61].

The supplementation of the diet with pistachios for 9 months in prediabetic participants had a significant lowering effect in fasting plasma glucose, fasting plasma insulin, and HOMA-IR (*p* < 0.001 for all), but only borderline significance in decreasing Wc, BW, and BMI [57]. Also, a short 4 week trial with a pecan-rich diet showed significant reductions in serum insulin and HOMA-IR compared to the control diet (*p* < 0.05) [41].

Similar to our findings, two meta-analysis of RCTs pointed out that nut consumption was related to a significant decrease in BW, BMI, and Wc [62,63].

One reason for these outcomes can be the fact that the metabolizable energy content, or energy available to the body, of several nuts is less than predicted by the Atwater factors: 5% less for pistachios [64], 16% less for cashews [65], 21% less for walnuts [66], and as much as 32% less in the case of almonds [67]. Also, despite 10% higher energy intake from peanuts in another trial, a less than expected increase in BW compared to the control was found [68].

The meta-analysis of Viguiliouk et al. [69] established that tree nut intake significantly lowered fasting blood glucose (*p* = 0.03) and HbA1c (*p* = 0.0003) compared with control diets, but no significant effects were detected for fasting insulin and HOMA-IR. The very recent meta-analysis from Tindall et al. [70] observed that consumption of tree nuts or peanuts significantly decreased fasting insulin and HOMA-IR with no significant effect on fasting blood glucose or HbA1c.

Positive associations between nut consumption and cardiometabolic biomarkers were seen in two cross-sectional studies reported in the literature in the last ten years, although the strength of the association is weaker in these types of studies, which look at a single point in time, compared to longitudinal and intervention studies. A cross-sectional study conducted on an elderly population at high CV risk, showed that nut intake decreased both Wc and BMI (*p*-trend < 0.005 in both) [71]. In the study of Jaceldo-Siegl et al. [72] obesity was 11% less likely in the high peanut/low tree nut group (odds ratio, OR = 0.89; 95% CI: 0.53–1.48), 37% less likely in the high tree nut/high peanut group (OR = 0.63; 95% CI: 0.40–0.99), and 46% less likely in the high tree nut/low peanut group (OR = 0.54; 95% CI: 0.34–0.88) (*p*-trend = 0.006) compared to low tree nut/low peanut group. Moreover, in a large US population cross-sectional survey, walnut consumers had a significantly lower prevalence of diabetes with lower levels of fasting blood glucose and HbA1c [73].

### 2.4. Nut Consumption Effect on Endothelial Function and Inflammation Markers

The studies, summarized in Table 4, proved that tree nut-enhanced diets can have beneficial effects on endothelial function or inflammatory markers [74,75,76,77,78,79]. Endothelial dysfunction, characterized by a decreased bioavailability of nitric oxide (NO), an endogenous vasodilator synthesized from the amino acid L-arginine, is linked with a greater risk of CV events [80]. Since tree nuts are a rich source of L-arginine, their intake might potentially improve endothelial dysfunction.

The trial of Ma et al. [74] on T2DM patients revealed that consumption of an ad libitum diet supplemented with 56 g walnuts daily for a period of 8 weeks significantly improved flow mediated dilation (FMD), a measure of endothelial function, as compared to a nut-free ad libitum diet. In a larger trial on overweight adults with visceral adiposity, the same research team found again that after daily ingestion of walnuts, FMD improved significantly (*p* = 0.019) as compared with the control diet, suggesting the potential for overall cardiac risk reduction [75]. Similarly, in hypercholesterolemic subjects, a diet with walnuts and walnut oil amended FMD (+34%) and reduced total peripheral resistance [81]. The study of Chen et al. [78] in patients with CAD established that almonds can also increase FMD, but did not significantly change C-reactive protein (CRP), tumor necrosis factor-alpha (TNF-α), or E-selectin compared to control. These results align with a meta-analysis published by Neale et al. [82] where nut intake had a significant effect on FMD (*p* = 0.0004). Equally, a recent meta-analysis of RCTs showed that nut consumption significantly increased FMD (*p* = 0.001) [83]. Bhardwaj et al. [84] noticed that walnut and almond diets, besides positively effecting FMD, can also decrease soluble vascular cell adhesion molecules.

While walnut and almond diets increased FMD, pistachios had no effect on this measure [38]. This fact was confirmed by the meta-analysis of Fogacci et al. [85] which did not notice a significant change in FMD, but the brachial artery diameter (BAD) significantly improved (*p* < 0.001) following pistachio consumption.

A very recent review [86] established that tree nut and peanut intake had the potential to improve vascular function, which was linked with increased risk of CVD.

In regard to inflammatory markers, the results of Liu et al. [76] proposed that the integration of almonds into a healthy diet in diabetic Chinese patients, as compared to the control diet, can decrease interleukin-6 (IL-6), TNF-α, and CRP. Both IL-6 and TNF-α are mediators of CRP synthesis in the liver. An increased IL-6 level was associated with insulin resistance, hyperglycemia, and T2DM [76]. The trial conducted by Sweazea et al. [77] concluded that almond intake, compared with control, significantly lowered CRP (*p* = 0.029) in diabetic U.S. patients, as well. In a recent study, Borkowski et al. [87] showed that walnuts can reduce the TNFα-mediated production of pro-inflammatory cytokines IL-6 and IL-8 in human adipocytes.

These findings are consistent with the results of a cross-sectional analysis which showed that higher tree nut and peanut intake was associated with decreased amounts of CRP and IL-6 [79]. However, the meta-analysis of Mazidi et al. [88] disclosed that nut consumption had no significant effect on CRP, IL6, TNF-α, and adiponectin, but significantly decreased leptin.

A low level of adiponectin, a protein with anti-inflammatory and anti-atherogenic properties secreted by adipocytes, is perceived as risk factor for CV events. An addition of 9 g of walnut oil or mixed plant oil in the diet increased adiponectin levels in T2DM subjects (+6.84% and +4.47%, respectively, compared to baseline) with borderline significance (*p* = 0.051) [89].

The favorable effects on inflammatory markers may be attributed to tree nuts, as a 12-week RCT on healthy mature adults with peanut intake failed to find any changes in inflammatory biomarkers [68].

The conclusion from these cohort and intervention studies is that the consumption of tree nuts and peanuts, with their high content of biologically active antioxidant compounds, should be encouraged for maintaining lower blood lipid counts and better biochemical and anthropometric parameters, in order to improve the prospect of cardiometabolic morbidity and mortality.

## 3. Association between Nuts and Cancer

Nutrition was demonstrated to have a causal and protective role in the progress of several types of cancer, the second leading cause of death worldwide [90].

A number of studies published in the last ten years have demonstrated the influence of nut consumption on cancer [91,92,93,94,95,96,97,98,99,100,101,102,103,104,105,106,107] (Table 5). Despite the fact that, in the observational studies, no causality could be proven, there were still several obvious strengths: prospective design for the majority of the studies, large population size, high retention rates with long-term follow-up, and adjustments for a multitude of other potential risk factors.

Several studies indicated that patients in the highest tree nut and peanut-intake group compared to the lowest intake group at any point in time during the study period were: 40%, 25%, and 14% less likely to die from total cancer, gastric noncardia adenocarcinoma, and lung cancer, respectively [16,97,98]. Also, they were 46%, 45%, and 47% less likely to die from esophageal squamous cell carcinoma, estrogen receptor negative breast cancer, and estrogen-progesterone receptor breast cancer, respectively [100,102]. Subjects having at least two servings of nuts per week had 0.68 times the risk of pancreatic cancer compared with subjects having nuts never or almost never [93]. The oil extracted from walnuts exhibited in vitro ability to reduce the viability of esophageal cancer cells, induced necrosis and cell cycle arrest, and displayed anticarcinogenic effect, thus it may present favorable effects in esophageal cancer in humans [108].

A recent prospective study showed that nut intake was not strongly associated with hepatocellular carcinoma but a significant inverse association with tree nut consumption was noted [106]. Higher intake of tree nuts and peanuts, but not peanut butter, was associated with a significantly reduced risk for small cell carcinoma (lung cancer subtype), after adjusting for smoking frequency and duration, and a non-significant decrease in lung cancer risk for men, results which have not been replicated in women [107].

The following case control studies also reported inverse associations between nut consumption and different types of cancer. In the highest intake group the outcomes were 57% and 28% less likely for prostate cancer and ovarian cancer, respectively [91,92]. Similarly, peanut consumption was linked with a lower risk of esophageal squamous cell carcinoma [103]. Lee et al. [104] noticed that odd ratios were 70% less likely for colorectal cancer in women and men, 60% (in women) and 77% (in men) less likely for rectal cancer, and 87% (in women) and 61% (in men) less likely for distal colon cancer, for the highest nut-intake group. Also, the results from a very recently published study suggest that, particularly among women, moderate to high nut intakes (2 to 5.5 servings/week) may be associated with a lower risk of colorectal adenomas, the precursor to most colorectal cancers [109]. Yang et al. [95] showed that the colorectal cancer risk for women who consumed tree nuts and peanuts ≥2 times per week was 13% lower compared to non-consumers, with a borderline statistical significance (*p*-trend = 0.06). Fadelu et al. [101] proved that higher total nut intake was linked with a significantly reduced incidence of cancer relapse and death in subjects with stage III colon cancer, but the benefit was limited to tree nut consumption.

These results confirm those of Casari and Falasca [110], who linked nut intake with a positive effect against cancer, and Aune et al. [22], who noticed a 15% decreased cancer risk in subjects eating 28 g of nuts daily compared to subjects who did not have nuts.

A new clinical trial showed that walnuts could alter tumor gene expression in women with confirmed breast cancer in ways expected to decrease cancer growth, delay proliferation, reduce metastasis, and increase cancer cell death [105]. Toledo et al. [111] indicated also that walnut-enriched diets could modulate breast cancer growth. These results are in accordance with those reported by Soriano-Hernandez et al. [112] where, in the group that consumed higher amounts of walnuts, almonds and peanuts, the breast cancer risk was between 55 and 67% less likely.

Higher nut consumption might influence cancer risk through its association with lower circulating levels of total cholesterol, CRP, IL-6, and insulin. Different compounds, such as flavonoids from nuts or their by-products might lower the risk of head and neck cancer [113] and could present chemopreventive properties and anticancer potential [114,115,116]. These bioactive antioxidant molecules can act alone or most likely in synergism to regulate the inflammatory response and immunological activity, prevent the development of prostaglandins or pro-inflammatory cytokines, and potentially inhibit cancer risk.

## 4. Association between Nuts and Cognitive Disorders

Inflammation-associated chronic pathologies, such as dementia, Parkinson’s disease (PD), or Alzheimer’s disease (AD), lead to one of the most unfavorable health problems in the elderly: age-related cognitive deterioration, a condition which may be prevented or delayed by modifiable lifestyle factors, including antioxidant diets [117].

Quite a few studies have examined the association between diets supplemented with nuts and cognitive performance [118,119,120,121,122,123,124] (Table 6).

In an elderly population, consumption of walnuts was related to better cognitive performance, mainly working memory, although the causality could not be inferred [120]. These results were consistent with another cross-sectional study that indicated a positive association between nut consumption and cognitive function in mature Chinese adults. Patients with mild cognition impairment symptoms had less tree nuts and peanuts in their diet compared to healthy subjects (*p* = 0.031) [125]. Similarly, positive relations between cognitive functions and nut intake were shown in the US population. Significant improvements in almost all cognitive test scores were noted among older adults who added walnuts in their diet [126].

The scores from two neuropsychological tests, the Mini-Mental State Examination (an indicator of cognitive impairment) and the Clock Drawing Test (a neuropsychological test which evaluates cognitive decline and dementia), were higher for subjects allocated to the nut-enhanced MD compared to the low-fat, nut-free diet group [121]. Comparable results were obtained by Valls-Pedret et al. [123]; in an older population, MD supplemented with tree nuts (walnuts, almonds, hazelnuts) was associated with improved cognitive functions. Also, a high consumption of tree nuts and peanuts was linked to better cognitive function at baseline and might reduce cognitive decline in mature adults [119]. Equally, O’Brien et al. [122] suggested that long-term tree nut and peanut intake was related to overall level of cognition but had no effect on cognitive decline.

A recent RCT demonstrated that peanut consumption could improve vascular and cognitive functions in overweight middle-aged subjects. Small artery elasticity, cerebrovascular reactivity, as well as measures of verbal fluency, processing speed, and short-term memory were all greater after higher intake of roasted, unsalted peanuts with skin [124]. Also, the addition of walnuts (15% of energy) to an ad libitum diet confirmed that regular nut consumption can delay the onset of age-related neurodegenerative disorders. Compared with the control, individuals in the walnut group reported significantly lower intake of animal protein, total carbohydrates, saturated fatty acids, and sodium, but significantly higher ingestion of vegetable protein, antioxidant *n*-3 and *n*-6 PUFAs [127].

Brain-derived neurotrophic factor (BDNF), a protein belonging to the neurotrophic family, controls axonal elongation, neurotransmitter release, growth, differentiation, and survival of presynaptic structure. While low plasma levels of BDNF could lead to the atrophy of specific brain areas in mammals such as the hippocampus and frontal cortex. Higher concentrations of BDNF provided by enhanced-nut MD were likely to prevent depression, memory loss, and cognitive decline [118]. It seems that *n*-3 PUFA, with its powerful antioxidant potential, is responsible for the increased levels of the BDNF signaling factor [128]. Blondeau et al. [129] noticed that alpha-linolenic acid (ALA), the plant-based *n*-3 PUFA, may increase BDNF, thus walnut intake can have a role in neuroprotection, neuroplasticity, and vasodilation of brain arteries.

Major depressive disorder (MDD) is a chronic disease where healthy dietary practices in combination with current treatments may prevent or delay its evolution. Increased consumption of nuts, seeds, vegetables, fruits, and legumes, with proven antioxidant and anti-inflammatory capacities, is the principal nutritional recommendation, with a key reminder that the beneficial effects are possible to come from wholesome nutritious diets rather than from individual nutrients [130]. Ali-Sisto et al. [131] showed that MDD is characterized by a decreased arginine level, an amino acid found in nuts and a precursor of NO, which is needed to modulate neuronal and vasodilation functions, to prevent oxidation of LDL-C, aggregation of platelets, or vascular inflammation, and inhibit oxidation in the central nervous system (CNS). MDD is associated with increased CV events, and the biological mechanism connecting MDD and CVD is apparently a chronic inflammation induced by a low level or bioavailability of arginine [131].

A study on Chinese adults demonstrated that, even after adjusting for potential confounders, the frequency of tree nut and peanut intake (more than 4 times per week) might be associated with a lower incidence of depressive symptoms [132]. Also, Arab et al. [133] reported that U.S. nut consumers, especially walnut eaters, had significantly lower depression scores as compared to no-nut consumers, and the difference was strongest among women.

Optimal dietary choices, such as increasing bioactive antioxidant compound intake through higher nut, fruit, and vegetable consumption, can improve endothelial function, decrease inflammatory biomarkers, protect neuronal and cell-signaling function, increase cognitive performance, and prevent or delay the onset of cognitive dysfunction during aging [134]. In anxiety-based psychopathology, replacing pro-inflammatory saturated fats with anti-inflammatory walnut oil might result in faster, more profound elimination of fear-based learning [135].

## 5. Other Possible Beneficial Association of Nuts

As the population of the world is getting older, a global priority for the aging population should be the maintenance of independence and freedom of movement. One of the hallmarks of aging and a major public health concern is the loss of mobility due to sarcopenia and dynapenia, or progressive loss of skeletal muscle mass and muscle strength, respectively. Another gerontological condition, described by physical inactivity, slow walking speed, fatigue, and weakness, is frailty [136].

In the elderly, both conditions, sarcopenia and frailty, are characterized by increased levels of pro-inflammatory cytokines, including TNF-α, IL-6, and CRP. Therefore, knowing its antioxidant and anti-inflammatory potential, dietary intake of food rich in monounsaturated fatty acids (MUFAs) and PUFAs is of particular interest for this age group [137]. One recent study mentioned that quantities of 2 to 5 g per day of marine *n*-3 PUFA, corresponding to approximately 20 to 50 g walnuts, is shown to reduce muscle wasting and augment the intracellular anabolic signaling, thus having beneficial effects for the prevention and treatment of sarcopenia in mature adults [138]. Also in this age group, malnutrition and sarcopenia frequently overlap. In order to overcome the loss of lean mass and meet the increased energy requirements, the recommended protein intake is higher (1.2–1.4 g/kg/day) than that of healthy adults [139]. Because the protein level is between 15 to 21% in most tree nuts and around 26% in peanuts, nuts should be included in healthy diets as plant food sources of protein [140].

Nutrition is a factor that could influence osteoarthritis (OA), the most dominant form of arthritis with limited treatment, mainly through symptom management [141]. As food impacts systemic lipid levels, high consumption of saturated fat is linked with higher levels of pro-inflammatory fatty acids, while diets rich in less-inflammatory MUFAs and PUFAs, lipids also found in tree nuts and peanuts (Table 7) may reduce cartilage degradation and OA progression [142].

In a group of postmenopausal women, MD enhanced with up to 20 g/day nuts was significantly associated with bone mineral density (*p* = 0.045), indicating that nuts may be beneficial in osteoporosis prevention [143].

Two biomarkers of aging, advanced glycation end products (AGEs) and length of telomeres, can be influenced by nut diets. AGEs, a complex class of compounds, can be formed in vivo, as part of normal metabolism, or acquired exogenously from unhealthy diets or environment. They can accumulate in tissues during aging. Data suggests that AGEs can have damaging effects in CVD, neurodegenerative diseases, T2DM, cancer, or sarcopenia [144,145,146]. It was shown that nut-enhanced MD may constitute a good instrument towards the inhibition of AGEs formation and absorption [145]. Telomeres, protecting DNA sequences at the end of the chromosomes, present a defensive mechanism against risk factors for a number of age-related diseases, including osteoporosis, CVD, CHD, T2DM, pulmonary fibrosis, AD, and cancer [147,148,149]. Several studies demonstrated that reactive oxygen species (ROS) can accelerate telomere attrition, induce DNA damage response, and senescence [150]. Sirtuins or the action of telomerase can counter telomere shortening. Among the environmental factors lessening telomere attrition are polyphenols, *n*-3 PUFA, or vitamin E, active antioxidant molecules [150]. A recent study linked regular nut consumption with telomere length and a significant reduction in cellular aging and biologic senescence [151]. Fiber, another valuable compound in nuts, can mediate longer telomeres and reduce biologic aging [152]. A two year trial conducted by Freitas-Simoes et al. [153] in an elderly population confirmed that supplying the diet with walnuts at 15% of daily energy (30 to 60 g/day) postponed leukocyte telomere attrition, potentially influencing the aging process.

Together with physical exercises, long-term daily intake of tree nuts and/or peanuts may contribute to maintaining the health of the skeletal system, muscle mass and strength, as well as the well-being of middle-aged and elderly population.

## 6. Phytochemicals and Mechanisms Responsible for the Beneficial Activity

Biologically active antioxidant compounds found in nuts can modulate essential physiological processes inside human bodies and influence key mechanisms of actions involved in aging and age-associated diseases [154].

Nut antioxidant polyphenols, the majority of which are found in the pellicle of nuts, can have anti-carcinogenic potential. They retard the initiation, differentiation, and proliferation of cancer cells, modulate signaling pathways related to cell survival, attenuate the growth of tumors, diminish angiogenesis and metastasis, and stimulate the expression of detoxification enzymes and antioxidants [155,156].

Some polyphenols are found in significant amounts in certain types of nuts, giving them specific biological actions. Thus, ellagic acid (EA), physiologically hydrolyzed from ellagitannins (ETs) abundant in walnuts, found also in pecans and pine nuts, could reduce adipocyte expansion and might be beneficial in the management of obesity and the metabolic complications related to obesity [157]. Another example is anacardic acid, a strong antioxidant polyphenol contained in cashew nut shells, which was shown to have anticancer potential, inhibited prostate tumor angiogenesis, cell proliferation, and prompted apoptosis [158,159,160].

Other polyphenols, found only in very small amounts in nuts, can contribute to beneficial health effects through their hormetic and/or synergistic actions with other polyphenols. In pistachios, the small amounts of genistein, (-) epigallocatechin-3-gallate (EGCG) or resveratrol can act synergistically through common or complementary action pathways with proven antioxidant and anti-aging activity. Thus, the flavonoid (isoflavone) genistein has demonstrated antioxidant, chemopreventive, and chemotherapeutic effects [161]. Growing evidence suggests that EGCG, also present in pecans and hazelnuts, can contribute to the anti-cancer potential [162]. It has an inhibitory proliferation effect on human pancreatic cancer cells [163]. In oral cancer, EGCG exerted an apoptotic therapeutic role, controlling cancer cell proliferation, and in breast cancer showed an anti-angiogenic effect [164]. Resveratrol, another antioxidant phytochemical also found in peanuts besides pistachios, proved to have neurogenesis activity and cancer chemoprevention potential [165,166]. Pterostilbene (PTS), a natural dimethylated analog of resveratrol, had the capability to significantly inhibit secretion of TNF-α and alter the cytokine production in IGROV-1 ovarian cancer cell line [167].

Melatonin, found in walnuts in quantities of 3.5 ± 1.0 ng/g, could be protective against CV damage and cancer initiation and propagation [168].

Selenium, a trace element supplied mostly by Brazil nuts, is associated with reduced risks for prostate cancer [169] and hepatocellular carcinoma [170].

Nuts are characterized as fatty foods, with total lipid content ranging from 46% in cashew to 76% in macadamia (Table 7). However, the healthy lipid profile of tree nuts and peanuts, mostly MUFAs and PUFAs and low or very low amounts of saturated fatty acids, is a key mechanism of action in the prevention of several age-related diseases [154,171].

Lipophilic bioactive compounds found in nuts can also influence the aging process. Among those compounds, phytosterols can reduce CV risk [172]. Increasing evidence recommends phytosterols for lowering LDL-C [173,174,175]. Phytosterols, being more hydrophobic than cholesterol, can dislocate cholesterol from intestinal micelles and reduce LDL-C absorption [176]. In combination with *n*-3 PUFAs, phytosterols show both complementary and synergistic lipid-lowering effects in hyperlipidemic mature adults [177].

Lipophilic isomers of vitamin E (tocopherols and tocotrienols), via their antioxidant properties, might inhibit the propagation of free radical damage in biological membranes and enhance immune functions [91]. Oxidation stress and inflammation, processes involved in the decline of cognitive function and neural capacity of the aging brain, can be reduced by tocols through their antioxidant and anti-inflammatory properties [178,179]. It was suggested that dietary intake of tocotrienols could be sufficient to support neuroprotection [180,181].

The lipophilic antioxidant phytochemicals, even in minute amounts, showed increased bioavailability and bioaccessibility, with their intestinal absorption being favored by the presence of lipids in tree nuts and peanuts. Lutein, the most abundant antioxidant carotenoid in the human retina and brain, can be found in pistachios [182]. Age-related macular degeneration (AMD), the primary cause of blindness and vision impairment in old age, can be amended or prevented by lutein [183]. Data indicated the significant impact macular pigment density, a biomarker of brain lutein, might also have on the brain health and cognition in the elderly by improving neurobiological efficiency, neural structure and efficacy, visual perception, and decision-making [184,185]. Recent scientific evidence showed that lutein could stop neuroinflammation, a pathological condition of many neurodegenerative disorders, diminish lipid peroxidation, and, by down-regulation of the NF-κB (the nuclear factor kappa-light-chain-enhancer of activated B cells) pathway, decrease the release of pro-inflammatory cytokines [186]. Compared to other sources, the amount of lutein found in pistachios is low, but as mentioned before, the intestinal absorption of lutein is enhanced by the presence of fatty acids in the tree nut.

Two mechanisms of actions, increased cholesterol efflux and improved endothelial function, favorably affected by whole walnuts and walnut oil, may answer in part the CV benefits of walnut consumption [187]. The favorable effect walnuts have on endothelial function could be credited to ALA, oxylipins (PUFA metabolites with a protective role in CVD and aging), polyphenols, L-arginine, and magnesium [176]. Walnut kernels provide ~9% ALA, while walnut oil provides ~10% ALA [81].

Similarly, ALA might be the factor for the decreased number of atherogenic small and dense LDL-C particles and increased number of large HDL-C particles noticed after walnut intake, as well as the reduction of detrimental lipid classes, such as ceramides and sphingomyelins, associated with CVD risk [188].

The synergistic influence exerted by MUFAs and PUFAs, antioxidant polyphenols and lipophilic compounds, and fiber in the modulation of specific miRNAs, have resulted in the improvement of insulin sensitivity via the PI3K-AKT signaling pathway in pre-diabetic and diabetic population [189].

In pathological conditions, such as AD, there is a diminished expression of glucose transporters, which apparently contributes to a reduced utilization of glucose in cognition-critical brain areas. However, transport and metabolism of ketone bodies (KBs), metabolites produced by the liver as alternative energy sources, are not affected in AD [190]. For that reason, periods of ketogenic diets (KDs) can possibly be effective preventive or treatment measures for neurological disorders [191]. We argue that nuts, due to their phytochemical profile (fat content between 49 and 75%, low amounts of carbohydrates, and high content of ketogenic amino acids including leucine) and strong antioxidant potential, can be part of KDs. Important actions of KDs are related to decrease oxidative stress and inflammatory activity and improve mitochondrial function [192].

As seen in this review, higher nut intake by mature adults was associated with a reduced risk of diabetes, CHD, CVD, several types of cancer, and cognitive disorders. For most of these outcomes, there were indications of nonlinear associations between tree nut and peanut consumption and decreased risk noticed up to an intake of around 15–20 g per day, or 4–5 servings per week, with no further decrease with higher intakes. One study revealed that walnuts had a beneficial effect against diabetes at about 5 grams per day, a little more than one serving per week, with again no additional results for greater intake amount. The intake of both tree nuts and peanuts was linked with reduced risks of diabetes, CHD, CVD, and cancer, as well as increased cognitive function and performance. Although, only tree nuts showed increase in flow-mediated dilation and decrease in LDL-C or certain biochemical and anthropometric parameters (fasting insulin, HOMA-IR, HbA1c, BW, Wc). The results of our study are in line with other analyses that have investigated the relationship between nut intake and chronic diseases [22,94].

## 7. The Association between Nut Intake and Gastrointestinal Microbiota

The relationship between gut microbiota (GM), diet, and healthy aging has been established in many studies [193,194,195]. Nutrition is a vital instrument in keeping a friendly microbiome, and this is more important in aging, when increased usage of medication can reduce healthy GM diversity and stability [196]. GM can impact CNS function, via gut-brain axis, and regulate the immune system [197,198]. Also, GM can be involved in several brain disorders (autism, PD, schizophrenia) [199]. Patients having PD, a high incidence neurodegenerative disorder among those over 60 years old, revealed pro-inflammatory bacteria in their gastrointestinal tract. Pathological by-products of these microorganisms could leak from the intestinal lumen in the enteric nervous system and aggregate into insoluble fibrils in the CNS [200,201].

Nut polyphenols were reported to increase the abundance of *Bifidobacterium* and *Lactobacillus* bacteria, probiotic strains related to significant lowering of CRP concentrations and increase in plasma HDL-C, cancer prevention, immune-modulation, as well as reductions of pathogenic *Clostridium* species and enteropathogens *Salmonella typhimurium* or *Staphylococcus aureus* [202,203]. Walnut ingestion increased the abundance of *Lactobacillus* [204], while decreasing microbial derived, proinflammatory LDL-C and secondary bile acids in healthy mature adults [205]. Similar results were achieved in a very recent 8-week long RCT, including 194 healthy individuals (mean age 63 years), where after 43 g/day walnut-enriched diet, the probiotic and butyric acid-producing species (*Ruminococcaceae* and *Bifidobacteria*) significantly increased (*p* < 0.02), while *Clostridium* species significantly decreased (*p* < 0.05) [206]. Also, pistachio and almond consumption via the prebiotic compounds may stimulate the growth of beneficial butyrate-producing bacteria and inhibit the development of pathogenic ones [207]. Holscher et al. [208] demonstrated that daily consumption of around 42 g almonds for at least 3 weeks can increase the abundance of *Roseburia* species, a favorable genus known to be negatively affected by age.

The favorable effect of walnut diets on BP may be linked to changes in the GM. As walnuts are not completely metabolized in the upper gastrointestinal tract, they provide substrate to the gut microbiome and may stimulate the production of short-chain fatty acids, including butyrate, which have been associated with normal BP management [209].

Human diet influences the relative abundance of bacterial communities present in the gastrointestinal tract [210]. A significant diversity and number of bacteria ensure a greater ability to resist metabolic changes and infections and constitute the prerequisite for a healthy status of the gut [202]. Consequently, a nut enhanced diet characterized by high antioxidant and anti-inflammatory activities can delay age-related microbiota changes and positively alter the microbial composition of the human GM with benefits for health.

## 8. Conclusions

Given that population aging has become a global trend, it is necessary to carefully evaluate age-associated diseases and identify strategies for promoting healthy lifestyle leading to healthy aging. Main goals should be the preservation of physical and cognitive functions, the maintenance of high standards of life quality and independence. Clearly, there is a need to design personalized recommendations to prevent, manage, or treat pathological conditions prevalent in the elderly.

As demonstrated in the present review, nuts, via their numerous biological active compounds (proteins, MUFAs and PUFAs, vitamins, minerals, fiber, polyphenols, phytosterols), have antioxidant and anti-inflammatory properties and might ensure cardioprotective benefits, safeguard against metabolic conditions, lower carcinogenic risk, help in cognitive disorders, or aid in sarcopenia and frailty. Just one bioactive compound cannot explain all these health benefits. It seems that antioxidant phytochemicals act synergistically to decrease the age-related oxidative stress and inflammation. Nuts, as complete functional foods, may positively adjust aging processes and play key roles in the relationship between lifespan and health span. Recent data favor the inclusion of robust antioxidant nuts in healthy diets of middle-aged and elderly population, a category steadily growing worldwide.

The scientific findings of our review stress the beneficial effects tree nut and peanut consumption can have in lowering risk factors related to several age-related diseases and highlight the importance of including nuts in healthy dietary plans. Moreover, the study has the potential to advance the perception of nuts as strong antioxidants between nutritionists, nurses, physicians, or general public and could be helpful in public health and health policy decisions.

## Figures and Tables

**Table 1:** Nut consumption and cardiometabolic morbidity and mortality.

Author, Year, Country [Ref]	Design	Subjects (F:M) Mean Age (Range)	Length of Study	Comparison Group	Intake of Nuts	Findings
Salas-Salvadó et al., 2011,2018 Spain [13]	RCT	418 (293:125) 67 (55–80) y	4 y	Control (low-fat diet *)	MD + 30 g/d nuts (15 g W, 7.5 g H, 7.5 g A)	↓ diabetes incidence, HR 0.47 (95% CI: 0.23–0.98) (vs. control)
Estruch et al., 2018, Spain [20]	RCT, Parallel, multicenter	2454 (1326:1128) 66.7 ± 6.1	4.8 y	Control (nut free diet)	MD + 30 g/d mixed nuts (15 g W, 7.5 g A, 7.5 g H)	↓ incidence of CV events (myocardial infarction, stroke, death from CV causes) (vs. control) HR 0.64 (95% CI: 0.47–0.88)
Pan et al., 2013, USA [14]	Prospective (NHS)	58,063 F 52–77 y	22 y		Tree nuts and peanuts (1) Never/rarely (2) <1 serving/wk (3) 1 serving/wk (4) 2–4 servings/wk (5) ≥5 servings/wk	↓ T2DM risk (*p*-trend < 0.001) for tree nuts and peanuts HR 0.80 (95% CI: 0.71–0.90) for (2) to (4) vs. (1) HR 0.74 (95% CI: 0.65–0.84) for (5) vs. (1) ↓ T2DM risk (*p*-trend = 0.002) for walnuts HR 0.76 (95% CI: 0.62–0.94) for (4),(5) vs. (1)
Ibarrola-Jurado et al., 2013, Spain [15]	Cross-sectional (PREDIMED)	7210 (4143:3067) 67 (55–80) y			Tree nuts and peanuts (1) <1 serving/wk (2) 1–3 servings/wk (3) >3 servings/wk	↓ prevalence of diabetes (3) vs. (1): OR 0.87 (95% CI: 0.78–0.99), *p*-trend = 0.043 ↓ prevalence of MS (3) vs. (1): OR 0.74 (95% CI: 0.65–0.85), *p*-trend < 0.001 ↓ prevalence of obesity (3) vs. (1): OR 0.61 (95% CI: 0.54–0.68), *p*-trend < 0.001
Guasch-Ferré et al., 2013, Spain [16]	Prospective (PREDIMED)	7216 (4145:3071) 67 y	4.8 y		Tree nuts and peanuts (1) none (2) 1–3 servings/wk (3) >3 servings/wk	↓ CV mortality (3) vs. (1) for walnuts HR 0.53 (95% CI: 0.29–0.98), *p*-trend = 0.047 ↓ CV mortality (3) vs. (1) for tree nuts (no walnuts) and peanuts HR 0.42 (95% CI: 0.20–0.89), *p*-trend = 0.031 ↓ total mortality risk (3) vs. (1) for tree nuts (walnuts included) and peanuts HR 0.61 (95% CI: 0.45–0.83), *p*-trend = 0.01
Hshieh et al., 2015, USA [17]	Prospective	20,742 M 67 y	9.6 y		Tree nuts and peanuts (1) <1 serving/mo (2) 1–3 servings/mo (3) 1 serving/wk (4) 2–4 servings/wk (5) ≥5 servings/wk	↓ CVD deaths (5) vs. (1) HR 0.74 (95% CI: 0.55–1.02), *p*-trend = 0.015
Guasch-Ferré et al., 2017, USA [18]	Prospective (a) NHS (b) NHS II (c) HPFS	(a) 76,364 F (b) 92,946 F (c) 41,526 M 56 y	28.7 y 21.5 y 22.5 y		Tree nuts and peanuts (1) Never/almost never (2) <1 time/wk (3) 1 time/wk (4) 2–4 times/wk (5) ≥5 times/wk	(5) vs. (1) for tree nuts and peanuts ↓ CVD-HR 0.86 (95% CI: 0.79–0.93, *p*-trend < 0.001) ↓ CHD-HR 0.80 (95% CI: 0.72–0.89, *p*-trend < 0.001) ≥2 times/wk tree nuts and peanuts ↓ 13–19% CVD risk ↓ 15–23% CHD risk
Larsson et al., 2018, Sweden [19]	Prospective	61,364 (28,453:32,911) 58 (45–83) y	17 y		Tree nuts and peanuts (1) none (2) 1–3 times/mo (3) 1–2 times/wk (4) ≥3 times/wk	↓ risk of atrial fibrillation for (4) vs. (1) (linear association) HR 0.82 (95% CI: 0.68–0.99), *p*-trend = 0.004 ↓ risk of heart failure for (3) vs. (1) (non-linear association) HR 0.80 (95% CI: 0.67–0.97), *p*-trend = 0.003 (fully adjusted models)
Liu et al., 2019, USA [21]	Prospective (NHS, HPFS)	16,217 (12,006:4211) 64.7–69.4 y	34 y 28 y		Tree nuts and peanuts (1) <1 serving/mo (2) <1 serving/wk (3) 1 serving/wk (4) 2–4 servings/wk (5) ≥5 servings/wk	(5) vs. (1) HRs ↓ CVD incidence, 0.83 (0.71–0.98), *p*-trend = 0.01 ↓ CHD incidence, 0.80 (0.67–0.96), *p*-trend = 0.005 ↓ CVD mortality, 0.66 (0.52–0.84), *p*-trend < 0.001 ↓ all-cause mortality 0.69 (0.61–0.77), *p*-trend < 0.001

* low-fat diet—all types of fat, from both animal and vegetable sources, reduced, but no fat-free foods. A—almonds; CHD—coronary heart disease; CI—confidence interval; CV—cardiovascular; CVD—cardiovascular diseases; F—women; H—hazelnuts; HR—hazard ratio; HPFS—Health Professionals Follow-Up Study; M—men; MD—Mediterranean diet; MS—metabolic syndrome; NHS—Nurses’ Health Study; OR—odds ratio; RCT—randomized controlled trial; RR—risk ratio; T2DM—type 2 diabetes mellitus; W—walnuts.

**Table 2:** Nut consumption and blood lipid levels in intervention studies.

Author, Year, Country [Ref]	Design	Subjects (F:M) Mean Age (±SD)	Length of Study	COMPARISON GROUP	Intake of Nuts	Findings
Li et al., 2011, Taiwan [31]	RCT, Crossover	20 (11:9) T2DM patients 58 y	12 wk	Control (diet without A)	56 g/d A	↓ T-C 6.0% (95% CI: 1.6–9.4), *p* ≤ 0.0025 ↓ LDL-C 11.6% (95% CI: 2.8–19.1), *p* ≤ 0.0117 ↓ LDL-C/HDL-C ratio 9.7% (95% CI: 0.3–20.9), *p* ≤ 0.0128 (vs. control)
Wu et al., 2014, Germany [32]	RCT, Crossover	40 (30:10) 60 ± 1 y	8 wk	Control (nut-free Western-type diet)	43 g/d W	↓ non-HDL-C (−10 ± 3 mg/dL, *p* = 0.025) ↓ apoB (−5.0 ± 1.3 mg/dL, *p* = 0.009) (vs. baseline)
Hernández-Alonso et al., 2015, Spain [33]	RCT, Crossover	54 (25:29) prediabetics 55 y	9 mo	Control (diet without pistachios)	57 g/d pistachio	↓ LDL-C (P) −28.07 nM (95% CI: −60.43 to 4.29) vs. baseline; *p* = 0.02 ↓ Non-HDL-C (P) −36.02 nM (95% CI: −77.56 to 5.52) vs. baseline; *p* = 0.04
Ruisinger et al., 2015, USA [34]	RCT, Parallel	48 (24:24) on statin therapy 60 y	4 wk	Control (diet without A)	100 g/d A	↓ non-HDL-C (113.4 ± 24.5 vs. 124.7 ± 20.8 mg/dL, *p* = 0.024) ↓ LDL-C (95.6 ± 23.9 vs. 104.3 ± 18.7 mg/dL, *p* = 0.068) ↓ TG (102.1 ± 38.3 vs. 115.0 ± 42.6 mg/dL, *p* = 0.068) (vs. control)
Jamshed et al., 2015, Pakistan [35]	RCT	150 (37:113) CAD patients 60 (32–86) y	12 wk	Control (diet without A)	10 g/d A before breakfast	↑ HDL-C (40 ± 6 vs. 33 ± 5 mg/dL) ↓ TG (118 ± 18 vs. 130 ± 20 mg/dL) (vs. baseline; *p* all < 0.05)
Njike et al., 2015, USA [36]	RCT, Parallel	112 (81:31) 55 y	6 mo	Control (diet without W)	56 g/d W	↓ T-C (−16.04 ± 27.34 mg/dL vs. baseline, *p* < 0.0001) ↓ LDL-C (−14.52 ± 24.11 mg/dL vs. baseline, *p* < 0.0001)
Huguenin et al., 2015, Brazil, [37]	RCT, Crossover	91 (44:47) hypertensive 62 y	12 wk	Control (nut-free diet)	13 g/d Granulated Brazil nut	↓ Ox LDL-C (60.68 ± 20.88 from 66.31 ± 23.59 U/L) (vs. baseline, *p* < 0.05)
Sauder et al., 2015, USA [38]	RCT, Crossover	30 (15:15) 56.1 ± 7.8 y	4 wk	Control (diet without pistachios)	pistachios (20% of total energy)	↓ T-C (4.00 ± 0.06 vs. 4.15 ± 0.06 mmol/L, *p* = 0.048) ↓ T-C/HDL-C (4.06 ± 0.08 vs. 4.37 ± 0.08, *p* = 0.0004) ↓ TG (1.56 ± 0.10 vs. 1.84 ± 0.10, *p* = 0.003) (vs. control)
Mah et al., 2017, USA [39]	RCT, Crossover	51 (31:20) 56 y	4 wk	Control (diet without cashews)	28–64 g/d cashews	↓ T-C 23.9% (95% CI: 29.3–1.7) vs. 0.8% (95% CI: 21.5–4.5) ↓ LDL-C 24.8% (95% CI: 212.6–3.1) vs. 1.2% (95% CI: 22.3–7.8) ↓ non-HDL-C 25.3% (95% CI: 28.6–2.1) vs. 1.7% (95% CI: 20.9–5.6%)(vs. baseline compared with control, *p* < 0.05)
Bamberger et al., 2017, Germany [40]	RCT, Crossover	194 (134:60) 63 ± 0.54 y	24 wk	Control (diet without W)	43 g/d W	↓ T-C (−9.5 vs. −2.2 mg/dL, *p* = 0.0003) ↓ LDL-C (−7.3 vs. −1.9 mg/dL, *p* = 0.0009) ↓ non-HDL-C (−9.4 vs. −1.5 mg/dL, *p* < 0.001) ↓ TG (−5.5 vs. 3.4 mg/dL, *p* = 0.0943) ↓ apoB (−6.8 vs. −0.9 mg/dL, *p* < 0.0001) (vs. control)
McKay et al., 2018, USA [41]	RCT, Crossover	26 (5:21) 59.7 (57–70) y	12 wk	Control (isocaloric, no pecans)	42.5 g/d pecans	↓ T-C (−8.89 ± 4.41, *p* = 0.056) ↓ LDL-C (−7.41 ± 3.85, *p* = 0.067)
Jenkins et al., 2018, Canada [42]	RCT, Parallel	117 (39:78) diabetics 62 y	3 mo	Controls (isocaloric (1) carbs diet and (2) carbs and nut diet)	75 g/d mixed nuts (tree nuts and peanuts)	↓ T-C −0.06 mmol/L (95% CI: −0.12 to −0.01), *p* = 0.026 ↓ non HDL-C −0.09 mmol/L (95% CI: −0.17 to −0.01), *p* = 0.026 ↓ apoB −0.09 g/L (95% CI: −0.16 to −0.02), *p* = 0.015 vs. control (1)
Bowen et al., 2019, Australia [43]	RCT	76 (31:45) 61 y	8 wk	Control (nut free diet)	56 g/d A	↓ TC/HDL-C ratio (in women, but not in men)

A—almonds; apoB—apolipoprotein B; CI—confidence interval; F—women; HDL-C—high density lipoprotein-cholesterol; LDL-C (P)—low density lipoprotein-cholesterol (particle); M—men; RCT—randomized controlled trial; SD—standard deviation; T2DM—type 2 diabetes mellitus; T-C—total cholesterol; TG—triglycerides; W—walnuts.

**Table 3:** Nut consumption and changes in biochemical and anthropometric parameters in intervention studies.

Author, Year, Country [Ref]	Design	Subjects (F:M) Mean Age (Range)	Length of Study	Comparison Group	Intake of Nuts	Findings
Li et al., 2011, Taiwan [31]	RCT, Crossover	20 (11:9) diabetics 58 y	12 wk	Control (diet without A)	60 g/d A	↓ fasting insulin 4.1% (95% CI: 0.9–12.5), *p* ≤ 0.0184 ↓ fasting glucose 0.8% (95% CI: 0.4–6.3), *p* ≤ 0.0238 ↓ HOMA-IR 9.2% (95% CI: 4.4–13.2), *p* ≤ 0.0039 (vs. control)
Cohen & Johnston, 2011, USA [54]	RCT	13 (6:7) diabetics 66 y	12 wk	Control (diet without A)	28 g/d A	↓ HbA1c (−4% vs. +1% for almond group vs. control), *p* = 0.045 ↓ BMI (−4% vs. 0% for almond group vs. control), *p* = 0.047
Damasceno et al., 2013, Spain [55]	RCT	169 (95:74) 67 (55–80) y	1 y	Baseline and Control (low-fat diet *)	MD + 30 g/d nuts (15 g W, 7.5 g H, 7.5 g A)	↓ Wc −5.1cm (95% CI: −6.8 to −3.4) vs. baseline; *p* ≤ 0.006 ↓VLDL-C −111 nmol/l (95% CI: −180 to −42) vs. baseline
Lasa et al., 2014, Spain [56]	RCT	191 (114:77) diabetics 67 (55–80) y	1 y	Baseline and Control (low-fat diet)	30 g/d nuts (15 g W, 7.5 g H, 7.5 g A)	↓ BW (−0.71 ± 2.41 kg vs. baseline of 75.2 ± 11.5 kg, *p* = 0.021) ↓ Wc (−4.84 ± 7.50 cm vs. baseline of 99.1 ± 8.96 cm, *p* = 0.001 for women)
Hernández-Alonso et al., 2014, Spain [57]	RCT, Crossover	54 (25:29) prediabetics 55 y	9 mo	Control (diet without pistachios)	57 g/d pistachio	↓ fasting glucose −5.17 mg/dL (95% CI: −8.14 to −2.19) vs. baseline; *p* < 0.001 ↓ fasting insulin −2.04 mU/mL (95% CI: −3.17 to −0.92) vs. baseline; *p* < 0.001 ↓HOMA-IR −0.69 (95% CI: −1.07 to −0.31) vs. baseline; *p* < 0.001 ↑ GLP−1 4.09 pg/mL (95% CI: 1.25–6.94) vs. baseline; *p* = 0.01
Rodríguez-Rejón et al., 2014, Spain [58]	RCT	2866 (1781:1085) non-diabetics 67 (55–80) y	1 y	Control (low-fat diet)	MD + 30 g/d nuts (15 g W, 7.5 g H, 7.5 g A)	↓ dietary GL −10.34 (95% CI: −12.69 to −8.00) ↓dietary GI −1.06 (95% CI: −1.51 to −0.62)
Chen et al., 2017, China [59]	RCT, Crossover	33 (20:13) diabetics 55 y	12 wk	Control (isocaloric diet no A)	60 g/d A	↓ fasting glucose 129.3 ± 25.6 (fast) vs. 132.8 ± 24.8 (pre) *p* = 0.011 ↓ HbA1c (%) 7.01 ± 0.62 (fast) vs. 7.18 ± 0.64 (pre) *p* = 0.043
Hou et al., 2018, China [60]	RCT	25 (10:15) diabetics 70 (40–80) y	3 mo	Compared to baseline	(1) Peanuts (60 g/d men, 50 g/d women) (2) A (55 g/d men, 45 g/d women)	↓ fasting glucose–in (1) and (2) ↓ postprandial 2-h blood glucose–in (1) and (2) (compared to baseline) (*p* < 0.05)
Jenkins et al., 2018, Canada [42]	RCT, Parallel	117 (39:78) diabetics 62 y	3 mo	Controls (isocaloric (1) carbs diet and (2) carbs and nut diet)	75 g/d mixed nuts (tree nuts and peanuts)	↓ HbA1c −2.0 mmol/mol (95% CI: −3.8 to −0.3), *p* = 0.026 vs. control (1)
McKay et al., 2018, USA [41]	RCT, Crossover	26 (5:21) 59.7 (57–70) y	12 wk	Control (isocaloric, no pecans)	42.5 g/d pecans	↓ fasting insulin (−2.00 ± 0.83 µIU/mL, *p* = 0.024) ↓ HOMA-IR (−0.51 ± 0.23, *p* = 0.037)

* low-fat diet—all types of fat, from both animal and vegetable sources, reduced, but no fat-free foods. A—almonds; BMI—body mass index; BW—body weight; CI—confidence interval; F—women; GI—glycemic index; GL—glycemic load; GLP-1—glucagon-like peptide-1; H—hazelnuts; HbA1c—hemoglobin A1c; HOMA-IR—Homeostasis Model of Assessment for insulin resistance; HR—hazard ratio; M—men; MD—Mediterranean Diet; RCT—randomized controlled trial; VLDL-C—very low density lipoprotein cholesterol; W—walnuts; Wc—waist circumference.

**Table 4:** Nut consumption and endothelial function and inflammatory biomarkers.

Author, Year, Country [Ref]	Design	Subjects (F:M)Mean Age (±SD)	Length of Study	Comparison Group	Intake of Nuts	Findings
Ma et al., 2010, USA [74]	RCT, Crossover	24 (14:10) 58.1 ± 9.2 y	8 wk	Control (diet without W)	56 g/d W	↑ FMD (2.2 ± 1.7 vs. 1.2 ± 1.6%, *p* = 0.04) (vs. control)
Katz et al., 2012, USA [75]	RCT, Crossover	46 (28:18) 57.4 ± 11.9 y	8 wk	Control (diet without nuts)	56 g/d W	↑ FMD 1.1% (95% CI: 0.2–2.0), *p* = 0.019 (vs. control)
Liu et al., 2013, Taiwan [76]	RCT, Crossover	20 (11:9) diabetics 58 y	12 wk	Control (diet without A)	56 g/d A	↓ IL-6 10.3% (95% CI: 5.2–12.6) ↓ TNF-α 15.7 % (95% CI: −0.3 to 29.9) ↓ CRP 10.3% (95% CI: −24.1 to 40.5), *p* = 0.0455 (vs. control)
Sweazea et al., 2014, USA [77]	RCT, Parallel	21 (12:9) 56.2 y	12 wk	Control (diet without A)	43 g/d A	↓ CRP in almond group vs. control (−1.2 vs. +4.33 mg/L, *p* = 0.029)
Chen et al. 2015, USA [78]	RCT Crossover	45 (26:18) 61.8 ± 8.6 y CAD patients	22 wk	Control (diet without A)	85 g/d A	↑ FMD, % (7.7 ± 3.3 vs. 8.3 ± 3.8%) (vs. control)
Yu et al., 2016, USA [79]	Cross-sectional (a)NHS (b)HPFS	(a) 3654 F; 59 y (b) 1359 M; 65 y	4 y		Tree nuts and peanuts (1) Never or almost never (2) <1 time/wk (3) 1 time/wk (4) 2–4 times/wk (5) ≥5 times/wk	↓ CRP–RR 0.90 (0.84–0.97) for (4) vs. (1); RR 0.84 (0.74–0.95) for (5) vs. (1) (*p*-trend = 0.006) ↓ IL-6–RR 0.88 (0.83–0.94) for (4) vs. (1); RR 0.88 (0.79–0.99) for (5) vs. (1) (*p*-trend = 0.016)

A—almonds; CI—confidence interval; CAD—coronary artery disease; CRP—C-reactive protein; F—women; FMD—flow-mediated dilation; NHS—Nurses’ Health Study; HPFS—Health Professionals Follow-Up Study; IL-6—interleukin-6; M—men; RCT—randomized controlled trial; RR—relative risk; SBP—systolic blood pressure; TNF-α—tumor necrosis factor-α; W—walnuts.

**Table 5:** Association between nut consumption and cancer.

Author, Year, Country [Ref]	Design	Subjects (F:M) Mean Age (Range)	Length of Intervention	Intake of Nuts	Findings
Hardman et al., 2019, USA [105]	RCT	10 women 55 y	2 to 3 wk	56 g/d walnuts	↓ growth and survival of breast cancer cells in walnut-diet group vs. control (no walnut)
Raimondi et al., 2010, Canada, [91]	Case-control study	394 men 69 y		Total nuts (g/d) (1) 0 (2) 0.1–1.2 (3) 1.3–3.0 (4) >3	↓ prostate cancer risk (4) vs. (1): OR 0.43 (95% CI: 0.22–0.85), *p*-trend = 0.01
Ibiebele et al., 2012, Australia [92]	Case-control study	2780 women 57 y		n-6 fatty acid (g) from total nuts (1) 0.13 (0.0–0.29) (2) 0.45 (0.29–0.68) (3) 1.48 (0.73–2.59) (4) 3.35 (2.59–25.9)	↓ ovarian cancer risk (4) vs. (1) OR 0.72 (95% CI: 0.57–0.92), *p*-trend = 0.02
Guasch-Ferré et al., 2013, Spain [16]	Prospective	7216 (4145:3071) high CV risk 67 y	4.8 y	Tree nuts and peanuts (1) none (2) 1–3 servings/wk (3) >3 servings/wk	↓ cancer mortality (3) vs. (1) for walnuts HR 0.46 (0.27–0.79), *p*-trend = 0.005 ↓ cancer mortality (3) vs. (1) for all nuts HR 0.60 (0.37–0.98), *p*-trend = 0.064
Bao et al., 2013, USA [93]	Prospective	75,680 women 59 y	30 y	Tree nuts and peanuts (1) never/almost never (2) 1–3 times/mo (3) 1 time/wk (4) ≥2 times/wk	↓ pancreatic cancer risk (*p*-trend = 0.01) RR 0.71 (95% CI: 0.51–0.99) for (3) vs. (1) RR 0.68 (95% CI: 0.48–0.96) for (4) vs. (1)
van den Brandt and Schouten, 2015, the Netherlands [94]	Prospective	120,852 (62,573:58,279) 61 y	10 y	Tree nuts and peanuts (1) 0 g/d (2) 0.1–5 g/d (3) 5–10 g/d (4) 10+ g/d	↓ cancer mortality (*p*-trend = 0.002) HR 0.82 (95% CI: 0.68–0.98) for (3) vs. (1) HR 0.79 (95% CI: 0.67–0.93) for (4) vs. (1)
Yang et al., 2016, USA [95]	Prospective	75,680 women 59 y	30 y	Tree nuts and peanuts (1) never/almost never (2) 1–3 times/mo (3) once/wk (4) ≥2 times/wk	↓ colorectal cancer risk (*p*-trend = 0.06), RR 0.87 (95% CI: 0.72–1.05) for (4) vs. (1)
Wang et al., 2016, USA [96]	Prospective	47,299 men 65 y	26 y	Tree nuts and peanuts (1) Never or almost never (2) <1 time/wk (3) 1 time/wk (4) 2–4 times/wk (5) ≥5 times/wk	↓ overall mortality after being diagnosed with non-metastatic prostate cancer (5) vs. (1) HR 0.66 (95% CI: 0.52–0.83), *p*-trend = 0.0005
Lee et al., 2017, Italy/USA [97]	EAGLE case-control study; NIH-AARP Diet and Health cohort study	3639—65 y 495,785—62 y	16 y	Tree nuts and peanuts 10 categories, ranging from “never” to ≥2 times per day; 3 categories for portion size	↓ lung cancer risk (highest vs. lowest nut intake) OR_EAGLE_ 0.74 (95% CI: 0.57–0.95), *p*-trend = 0.017 HR_AARP_ 0.86 (95% CI: 0.81–0.91), *p*-trend < 0.001
Hashemian et al., 2017, USA [98]	Prospective	566,407 (59.6% men) 63 (50–71) y	15.5 y	Tree nuts, peanuts, peanut butter Expressed in g/1000 kcal: (C0) 0 (C1) 0.11 (0.05, 0.16) (C2) 0.51 (0.36, 0.68) (C3) 2.20 (1.35, 4.12)	↓ gastric noncardia adenocarcinoma risk (C3) vs. (C0): HR 0.73 (95% CI: 0.57–0.94, *p*-trend 0.004) for tree nuts and peanuts HR 0.75 (95% CI: 0.60–0.94, *p*-trend 0.02) for peanut butter
Nieuwenhuis and van den Brandt 2018, the Netherlands [99]	Prospective	120,852 (62,573:58,279) 62 (55–69) y	20.3 y	Tree nuts and peanuts: (1) non-consumers (2) 0.1–5 g/d (3) 5–10 g/d (4) >10 g/d	↓ pancreatic cancer risk in men—(3), (4) vs. (1) HR 0.53 (95% CI: 0.28–1.00), *p*-trend = 0.047
Nieuwenhuis and van den Brandt 2018, the Netherlands [100]	Prospective	120,852 (62,573:58,279) 62 (55–69) y	20.3 y	Tree nuts and peanuts: (1) non-consumers (2) 0.1–5 g/d (3) 5–10 g/d (4) >10 g/d	↓ esophageal squamous cell carcinoma risk(4) vs. (1) HR 0.54 (95% CI: 0.30–0.96), *p*-trend = 0.050
Fadelu et al., 2018, USA [101]	Prospective	826 patients with stage III colon cancer	6.5 y	Tree nuts and peanuts (1) none (2) ≥2 servings/wk	↑ disease-free survival (2) vs. (1) HR 0.58 (95% CI: 0.37–0.92), *p*-trend = 0.03 ↑ overall survival (2) vs. (1) HR 0.43 (95% CI: 0.25–0.74), *p*-trend = 0.01 ↓ cancer recurrence and mortality
van den Brandt and Nieuwenhuis 2018, the Netherlands [102]	Prospective	62,573 women 61 (55–69) y	20.3 y	Tree nuts and peanuts: (1) non-consumers (2) 0.1–5 g/d (3) 5–10 g/d (4) >10 g/d	↓ (ER -) breast cancer risk (4) vs. (1) HR 0.55 (95% CI: 0.33–0.93), *p*-trend = 0.025 ↓ ER–PR breast cancer risk (4) vs. (1) HR 0.53 (95% CI: 0.29–0.99), *p*-trend = 0.037
Zhao et al., 2018, China [103]	Case-control study	444 (152:292) 59 (40–69) y		Peanuts: (1) <1/mo (2) 1–3 times/mo (3) 1–3 times /wk (4) 4–6 times/wk	↓ esophageal squamous cell carcinoma risk(4) vs. (1) OR 0.31 (95% CI: 0.16–0.59), *p*-trend < 0.001
Lee et al., 2018, Korea [104]	Case-control study	2769 (894:1875) 57 (48–66) y		Tree nuts and peanuts (1) None (2) <1 serving (15g)/wk (3) 1–3 servings/wk (4) ≥3 servings/wk	↓ colorectal cancer risk (F,M) (4) vs. (1) OR 0.30 (95% CI: 0.20–0.45), *p*-trend < 0.001 ↓ distal colon cancer risk (4) vs. (1) OR 0.13 (95% CI: 0.04–0.48), *p* < 0.001 for F OR 0.39 (95% CI: 0.19–0.80), *p* = 0.004 for M ↓ rectal cancer risk (4) vs. (1) OR 0.40 (95% CI: 0.17–0.95), *p* = 0.006 for F OR 0.23 (95% CI: 0.12–0.46), *p* < 0.001 for M
Sui et al., 2019, USA [106]	Prospective, NHS and HPFS	88,783 women 51,492 men 63 y	27.9 y	Tree nuts, servings/wk (1) 0.01 (2) 0.23 (3) 1.25	↓ hepatocellular carcinoma (3) vs. (1) HR 0.64 (95% CI: 0.43–0.95), *p*-trend = 0.07
Nieuwenhuis and van den Brandt 2019, the Netherlands [107]	Prospective	120,852 (62,573:58,279) 62 (55–69) y	20.3 y	Tree nuts and peanuts: (1) non-consumers (2) 0.1–5 g/d (3) 5–10 g/d (4) >10 g/d	↓ small cell carcinoma (lung cancer subtype) in men—(4) vs. (1) HR 0.62 (95% CI: 0.43–0.89), *p*-trend = 0.024 ↓ lung cancer risk in men (non-significantly)

AARP—American Association of Retired Persons; CI—confidence interval; CV—cardiovascular; EAGLE—the Environment And Genetics in Lung cancer Etiology; (ER -)—estrogen receptor negative; F—women; HPFS—Health Professionals Follow-up Study; HR—hazard ratio; M—men; n-6—omega 6; NHS—Nurses’ Health Study; NIH—National Institutes of Health; OR—odds ratio; PR—progesterone receptor; RCT—randomized controlled trial; RR—relative risk.

**Table 6:** Association between nut consumption and cognitive disorders.

Author, Year, Country [Ref]	Design	Subjects (F:M) Mean Age (Range)	Length of Intervention	Comparison Group	Intake of Nuts	Findings
Sánchez-Villegas et al., 2011, Spain [118]	RCT	152 (76:76) 68 y	3 y	Control (low-fat diet *)	MD + 30 g/d nuts (15 g W + 15 g A)	↓ risk for low plasma BDNF values OR 0.22 (95% CI: 0.05–0.90, *p* = 0.04) vs. control
Martínez-Lapiscina et al., 2013, Spain [121]	RCT, multicenter	522 (289:233) 67 y	6.5 y	Control (low-fat diet *)	MD + 30 g/d nuts (15 g W, 7.5 g H, 7.5 g A)	↑ cognition ↑ MMSE 0.57 (95% CI: 0.11–1.03, *p* = 0.015) vs. control ↑ CDT 0.33 (95% CI: 0.003–0.67, *p* = 0.048) vs. control
Valls-Pedret et al., 2015, Spain [123]	RCT	334 (170:164) 67 (55–80) y	4.1 y	Control (low-fat diet *)	MD + 30 g/d nuts (15 g W, 7.5 g H, 7.5 g A)	↓ age-related cognitive decline ↑ memory composite 0.09 (95% CI: −0.05 to 0.23, *p* = 0.04) vs. control ↑ frontal cognition composite 0.03 (95% CI: −0.25 to 0.31, *p* = 0.03) vs. control
Barbour et al., 2017, Australia [124]	RCT, Crossover	61 (32:29) 65 y	12 wk	Control (nut free diet)	56–84 g peanut/d	↑ cognitive functions (short-term memory, verbal fluency, processing speed) vs. control
Nooyens et al., 2011, the Netherlands [119]	Prospective	2613 (1325:1288) 55 (43–70) y	Ongoing since 1995		Tree nuts and peanuts 5 quintiles of nut consumption	↑ cognitive function at baseline ↓ cognitive decline: memory (highest vs. lowest nut intake, *p* = 0.03); global cognitive function (highest vs. lowest nut intake, *p* = 0.02)
Valls-Pedret et al., 2012, Spain [120]	Cross-sectional	447 (233:214) 67 (55–80) y			30 g W/d	↑ cognitive function (working memory, *p* = 0.039)
O’Brien et al., 2014, USA [122]	Prospective	15,467 women 74 y	6 y		Tree nuts and peanuts (1) never, <1/mo (2) 1–3/mo (3) 1/wk (4) 2–4/wk (5) 5/wk	↑ cognitive performance ↑ cognition (4), (5) vs. (1)

* low-fat diet—all types of fat, from both animal and vegetable sources, reduced, but no fat-free foods. A—almonds; BDNF—brain-derived neurotrophic factor; CDT—Clock Drawing Test; CI—confidence interval; F—women; H—hazelnuts; M—men; MD—Mediterranean diet; MMSE—Mini-Mental State Examination; OR—odds ratio; RCT—randomized controlled trial; W—walnuts.

**Table 7:** Average fat composition of nuts [3,139].

Mean Value (g/100 g)	Almond	Brazil Nut	Cashew	Hazelnut	Macadamia	Pecan	Pine Nuts	Pistachio	Walnut	Peanut
Total fat	49.9	67.1	43.8	60.7	75.8	72.0	68.4	45.3	65.2	49.2
SFA	3.8	16.1	7.8	4.5	12.1	6.2	4.9	5.9	6.1	6.8
MUFA	31.6	23.9	23.8	45.7	58.9	40.8	18.8	23.3	8.9	24.4
PUFA	12.3	24.4	7.8	7.9	1.5	21.6	34.1	14.4	47.2	15.6
(MUFA + PUFA)/SFA	11.6	3.0	4.1	11.9	5.0	10.1	8.8	6.4	9.2	5.9

MUFA—monounsaturated fatty acids; PUFA—polyunsaturated fatty acids; SFA—saturated fatty acids.

## Abbreviations

AD	Alzheimer’s disease
AGEs	advanced glycation end products
AMD	age-related macular degeneration
apoB	apolipoprotein B
BDNF	brain-derived neurotrophic factor
BMI	body mass index
BP	blood pressure
BW	body weight
CAD	coronary artery disease
CHD	coronary heart disease
CI	confidence interval
CNS	central nervous system
CRP	C-reactive protein
CVD	cardiovascular diseases
EA	ellagic acid
EGCG	(−)-epigallocatechin-3-gallate
ER	estrogen receptor
ETs	ellagitannins
FMD	flow-mediated dilation
GLUTs	glucose transporters
GM	gut microbiota
HbA1c	hemoglobin A1c
HDL-C	high density lipoprotein-cholesterol
HOMA-IR	Homeostatic Model Assessment—Insulin Resistance
HR	hazard ratio
IHD	ischemic heart disease
IL-6	interleukin 6
KBs	ketone bodies
KDs	ketogenic diets
LDL-C	low density lipoprotein-cholesterol
MDD	major depressive disorder
MD	Mediterranean diet
MS	metabolic syndrome
MUFAs	monounsaturated fatty acids
NO	nitric oxide
OR	odds ratio
PD	Parkinson’s disease
PTS	pterostilbene
PUFAs	polyunsaturated fatty acids
RCT	randomized controlled trial
ROS	reactive oxygen species
RR	relative risk
T-C	total cholesterol
T2DM	type 2 diabetes mellitus
TG	triglycerides
TNF-α	tumor necrosis factor-α
VLDL-C	very-low-density lipoprotein cholesterol
Wc	waist circumference
